# Respiratory infection by *Corynebacterium striatum*: epidemiological and clinical determinants

**DOI:** 10.1002/nmi2.48

**Published:** 2014-06-27

**Authors:** F Renom, M Gomila, M Garau, M d C Gallegos, D Guerrero, J Lalucat, J B Soriano

**Affiliations:** 1 Respiratory Department, Hospital Joan MarchBunyola, Balearic Islands, Spain; 2 Microbiologia, Departament de Biologia, Universitat de les Illes Balears, Institut Mediterrani d'Estudis Avançats (CSIC-UIB)Palma de Mallorca, Balearic Islands, Spain; 3 Microbiology Service, Hospital Son LlàtzerPalma de Mallorca, Balearic Islands, Spain; 4 Programme of Epidemiology and Clinical Research, Fundació Caubet-CIMERA Illes Balears, International Centre for Advanced Respiratory MedicineBunyola, Balearic Islands, Spain

**Keywords:** Advanced chronic obstructive pulmonary disease, *Corynebacterium striatum*, epidemiology, genotype, multidrug-resistance, respiratory infection

## Abstract

The increasing prevalence of advanced chronic respiratory disease, with frequent exposure to broad-spectrum antibiotics for repeated and prolonged hospitalizations, favours the emergence of nosocomial respiratory infection by Gram-positive bacteria, such as outbreaks of *Corynebacterium striatum*. There is little evidence about patterns of respiratory infection, transmission and adaptive ability of this pathogen. Seventy-two *C. striatum* isolates from 51 advanced respiratory patients, mainly chronic obstructive pulmonary disease, were studied during 38 months. Patients were 74.8 ± 8.6 years old and 81.9% were men, who had required an average of 2.2 hospitalizations and 63.5 days in the hospital in the previous year. Of 49 isolates from 42 patients we were able to identify 12 clones by multilocus sequence analysis (MLSA), nine phenotypic variants and 22 antibiotic susceptibility patterns, and we determined their clinical and epidemiological determinants. MLSA allows identification of the existence of nosocomial outbreaks by transmission of the same or different clones, the persistence of the same clone in the environment or in patient airways for months. The study showed the high variability and adaptive capacity of the isolates, the antibiotic multidrug-resistance in all of them, and their contribution to a high morbidity and mortality (41%) during the study period.

## Introduction

*Corynebacterium* species are found colonizing skin, other tissues, and in the environment 1,2; they are considered normal flora and not potentially pathogenic. But in 1997 Funke *et al*. 3 described a massive increase in the number of publications related to this genus, which were attributed to the increase of immunocompromised patients susceptible, to improved microbiological diagnoses, and to a precise taxonomic classification that allows the correct identification of different species with different clinical expression.

In the last decades, in addition to *Corynebacterium diphtheriae*, the pathogenicity among *Corynebacterium* spp. has been reported associated with *Corynebacterium amycolatum* 4,5, *Corynebacterium jeikeium*, *Corynebacterium macginleyi*, *Corynebacterium urealyticum*, *Corynebacterium pseudodiphtheriticum* and, less frequently with *Corynebacterium xerosis* 4,6–8. *Corynebacterium striatum* has been reported colonizing prostheses, catheter tips, and ventilator and feeding tubes, and it has been also identified as causative in cases of endocarditis, sepsis and bacteraemia 9–11.

Until 1993 there were only three individual case reports of confirmed respiratory infections by *C. striatum* 12–14. Since then, numerous individual cases and various nosocomial infectious outbreaks of *C. striatum* have been reported 15–21 mostly in patients with chronic diseases requiring frequent and prolonged hospitalizations with repeated exposure to antibiotics against Gram-negative bacteria, organic obstructive disorders, or exposed to invasive procedures that disrupted the skin barrier. Most reported *C. striatum* infections have been found in respiratory samples, the vast majority of strains being multidrug-resistant.

The study of nosocomial infections and outbreaks is fundamental to have reliable methods for the identification and typing of the bacteria responsible. It allows further efforts to prevent and control these events, and to reduce individual and population burden. There are several available fast and affordable methods that allow a good identification of species and useful for the identification of an outbreak, but understanding the mechanisms and transmission circumstances during an outbreak requires a molecular study of the isolates. Several studies have tried to accomplish this objective 16,19,21,22, but none of them employed a methodology for identification and typing of strains until the study of Gomila *et al*. 23. In that study, isolates of *C. striatum* were analysed using different approaches, ribotyping, phenotype, matrix-assisted laser desorption/ionization time-of-flight mass spectroscopy, and multilocus sequence analysis (MLSA). They demonstrated that isolates of *C. striatum* were best identified using gene-based molecular methods.

The main aim of this prospective study is to explore the epidemiological and pathological circumstances of a group of patients susceptible to be infected by *C. striatum*, managed in a respiratory ward. We determined the genetic identity of *C. striatum* strains isolated in cultures of biological samples obtained during infectious respiratory exacerbations. We aimed to study the clinical and environmental determinants, the transmission mechanisms between patients, their adaptive ability and variability in phenotype and susceptibility to antibiotics, its ability to persist in the environment and respiratory airways, and its impact on morbidity and mortality in this group of patients.

## Material and Methods

### *Corynebacterium striatum* culture collection

The Hospital Joan March, in Bunyola, Mallorca, Spain, is a secondary healthcare centre that hosts a convalescence and rehabilitation department, with a 26-bed ward. It aims to deliver care to patients with severe, chronic respiratory disease referred from tertiary-care hospitals within its catchment area.

Seventy-one *C. striatum* isolates from cultures of respiratory samples, and one from a dermal ulcer, from 51 patients with advanced respiratory disease seen in the hospital, were obtained during 38 months (from May 2006 to June 2009), and we explored their clinical and epidemiological determinants. Forty-nine of these *C. striatum* isolates, from 42 patients, could be recovered with the aim of analysing their antibiotic susceptibility, phenotype and genotype.

All respiratory samples were obtained during respiratory infectious exacerbations that fulfilled GOLD criteria 24, and according to the criteria of Anthonisen *et al*. 25 were of potential infectious origin. Specimens were cultured on Columbia agar with 5% sheep blood (bioMérieux, Marcy l'Etoile, France). Before processing, all samples were Gram-stained so as to discard the samples not representative of the lower respiratory tract and/or contaminated with microbiota from the upper respiratory tract according to the Murray and Washington criteria 26. The clinical relevance of *C. striatum* isolation from respiratory samples was based on their correct identification, their abundance, their isolation as a single microorganism or their predominance when found in association, and the repetition of positivity 1. The processing and incubation of plates was performed following routine laboratory conditions. All isolates were stored at −80°C for future study.

### Phenotypic and antibiotic susceptibility, and molecular analysis

All information about phenotype, genotype and antibiotic susceptibility was consistent with our previous work on 49 recovered isolates of the total 72 23, and they were labelled with alphabetical codes. Coding is described in Table S1 (see the Supporting information). From the molecular analysis only the internally transcribed spacer 1 (ITS1) region and the *gyrA* and *rpoB* genes were used, as they were the most useful for discriminating between strains due to their variability.

### Isolates versus temperature and humidity

Given that *C. striatum* is an environmental bacterium, temperature and humidity were recorded on the date of obtaining each isolate. Information was obtained from the official weather station in the enclosed area of the hospital. For this environmental analysis, the 43 isolates of *C. striatum* from a previous outbreak that occurred in our hospital between January 2004 and June 2005 were also considered 20.

### Statistical analysis

Statistical analyses were performed using the SPSS v.15.0 software. All data were quality controlled and comprehensive tabulations with mean, standard deviation, and ranges for quantitative variables, and counts and percentage of all quantitative variables, were presented.

## Results

Seventy-two *C. striatum* isolates were obtained during 38 months, mainly from cultures of respiratory samples of 51 patients, 81.9% men, with a mean age of 74.8 years. They suffered an average of 2.2 hospitalizations and 63.5 hospital days in the year before obtaining a positive culture. Overall, 84.7% had a diagnosis of chronic obstructive pulmonary disease, with a mean ± SD forced expiratory volume (FEV1%) of 39.4 ± 16.6%. Forty-nine percent of patients died during follow up. A code was assigned to each patient, from 1 to 51, sequentially by order of entry, and in patients who showed more than one isolate, a letter was added to the patient number in alphabetical order, from ‘a’ to ‘c’ (Table1).

**Table 1:** Chronological list of isolates analysed by molecular methods, with location, number of patient, genotype, phenotype and antibiotype codes, culture results, number of hospitalizations and number of days of hospitalizations per year previous to an isolate and date of death

Culture date (MM/DD/YY)	Room > transfer	Patient	Culture results^a^	ST	GC	PC	AC	FEV1%	No. hosp/year	No. days/year	Exitus date (MM/YY)
03/03/06	107	**1**	1	2	A	b	m	45%	3	54	
03/07/06	125	2	1	2	A	a	f	37%	3	179	08/06
03/16/06	AMB^b^	**3**	2	5	F	a	v	21%	1	15	
03/21/06	113	4	1	2	A	a	a	29%	1	30	
04/26/06	117	5	1	NR	NR	NR	NR	25%	5	71	06/08
05/05/06	AMB	**1a**^d^	1	NR	NR	NR	NR	45%	5	66	09/07
05/10/06	121	6	2	NR	NR	NR	NR	NA	3	69	03/07
05/18/06	AMB	7	4	6	K	a	d	18%	0	0	
05/25/06	101	8	1	1	D	a	k	25%	6	40	06/08
06/22/06	105	**3a**	2	1	D	a	k	21%	1	30	
07/06/06	119 > 213	**9**	1	NR	NR	NR	NR	43%	1	90	
10/17/06	129	**9a**	1	NR	NR	NR	NR	43%	1	190	
11/20/06	AMB > 101 > 125	10	1	7	M	a	h	30%	4	38	05/08
11/29/06	AMB	11	1	8	G	a	y	46%	0	0	
12/13/06	129	**9b**	2	NR	NR	NR	NR	43%	1	240	01/07
12/20/06	117	12	3	2	A	a	w	14%	2	20	01/07
12/20/06	109	**13**	4	1	D	a	m	NA	4	97	
01/02/07	111	14	1	NR	NR	NR	NR	42%	2	72	
01/26/07	117 > 103	15	1	2	A	a	a	36%	4	99	02/07
02/02/07	133	**13a**	2	NR	NR	NR	NR	NA	4	144	02/07
02/05/07	101	**16**	1	NR	NR	NR	NR	30%	1	36	
02/06/07	101	**16a**	1	2	A	c	b	30%	1	37	
02/07/07	101	**16b**	1	2	A	c	b	30%	1	38	
02/13/07	121	17	1	NR	NR	NR	NR	NA	1	4	08/08
02/20/07	107 > 105 > AMB	**18**	1	3	E	d	c	33%	1	27	
02/23/07	123 > AMB	19	2	2	A	c	o	50%	2	40	
02/28/07	117 > 115	20	1	4	B	c	a	32%	4	40	
03/09/07	115	21	1	4	B	e	a	27%	1	35	04/07
03/13/07	121	22	1	5	F	e	c	97%	2	62	06/08
03/20/07	123	23	2	4	B	c	a	14%	3	105	01/08
04/04/07	117	**18a**	1	3	E	d	c	33%	2	41	
04/14/07	103 > HSLL^c^	**24**	1	NR	NR	NR	NR	31%	1	8	
04/23/07	121 > 129	25	4	3	E	d	d	68%	2	31	08/07
05/14/07	418 HSLL>123	**24a**	2,3	NR	NR	NR	NR	31%	1	36	
05/15/07	131	26	1	NR	NR	NR	NR	34%	1	120	06/09
06/07/07	123	**24b**	1	2	A	c	f	31%	1	55	
06/13/07	AMB	**18b**	1	NR	NR	NR	NR	33%	3	51	
06/19/07	109	**27**	1	1	D	a	l	31%	4	40	
06/19/07	111	28	1	4	B	a	a	48%	1	64	12/07
06/20/07	109	**27a**	1	NR	NR	NR	NR	31%	4	41	
07/18/07	129	29	1	NR	NR	NR	NR	28%	3	36	
07/19/07	AMB	30	1	1	D	a	a	45%	1	12	
07/24/07	107	31	2	4	B	a	a	30%	2	107	06/09
07/25/07	123	**24c**	2	2	A	c	f	31%	1	123	
09/20/07	119	**32**	1	NR	NR	NR	NR	57%	2	51	
09/25/07	117	**33**	1	NR	NR	NR	NR	67%	1	17	
10/22/07	127	34	1	4	B	a	b	48%	1	20	12/07
01/04/08	105	35	2	9	H	k	t	33%	4	86	07/08
01/11/08	AMB	**3b**	2	NR	NR	NR	NR	21%	1	33	
02/05/08	AMB	**3c**	2,3	5	F	a	h	21%	1	33	04/08
02/19/08	131	**33a**	2	1	D	a	s	67%	3	67	04/08
02/29/08	121	36	2	2	A	c	b	19%	1	90	06/08
03/26/08	119	**32a**	3,4	5	F	a	c	57%	2	98	10/08
04/01/08	123	37	2	3	E	g	d	34%	5	137	
04/09/08	121	38	1	4	B	b	b	60%	4	46	
05/12/08	107	39	1	4	B	b	b	59%	4	74	
05/15/08	131	40	2,4	4	B	f	z	30%	3	87	
10/09/08	105	41	1	4	B	a	l	NA	5	70	
11/28/08	204	42	1	10	L	a	r	NA	2	50	
12/01/08	103	43	1	NR	NR	NR	NR	57%	1	38	
01/13/09	230	44	1	11	C	b	p	NA	1	39	
02/04/09	103	**45**	1	11	C	a	e	57%	1	11	
02/18/09	112 HG[Table-fn tf1-4]	**46**	2	8	G	b	g	52%	1	85	
02/19/09	112 HG	**46a**	2	8	G	b	g	52%	1	86	
02/23/09	226	47	4	11	C	b	n	NA	1	180	02/09
02/25/09	119	**48**	3	NR	NR	NR	NR	57%	1	32	
02/25/09	103	**45a**	4	11	C	b	e	19%	3	88	
03/10/09	131	49	4	11	C	b	g	76%	4	43	
03/11/09	103	**45b**	3	11	C	h	e	57%	1	46	
03/31/09	119	**48a**	4	NR	NR	NR	NR	19%	3	88	
05/12/09	111	50	1	NR	NR	NR	NR	61%	4	134	
06/22/09	117	51	4	15	J	c	x	33%	2	23	

In bold and with different lowercase letters the patients that appear more than once are indicated; i.e. **1a** = patient no. 1, second isolate. AC, antibiotype code; GC, genotype code; NA, not available; NR, unrecovered; PC, Phenotype code; ST, sequence type.

a Culture results: 1, *C. striatum* as pure culture; 2, *C. striatum* plus *Pseudomonas aeruginosa*; 3, *C. striatum* plus *Stenotrophomonas maltophilia*; 4, *C. striatum* plus other microorganisms.

b Outpatient.

c Hospital Son Llàtzer.

d Hospital General.

### Bacteria description

Of 72 specimens, 71 corresponded to respiratory samples: 69 of spontaneous sputum, one bronchoaspirate, and one tracheal aspirate, while the other specimen was from a dermal ulcer smear.

**Figure 1:**
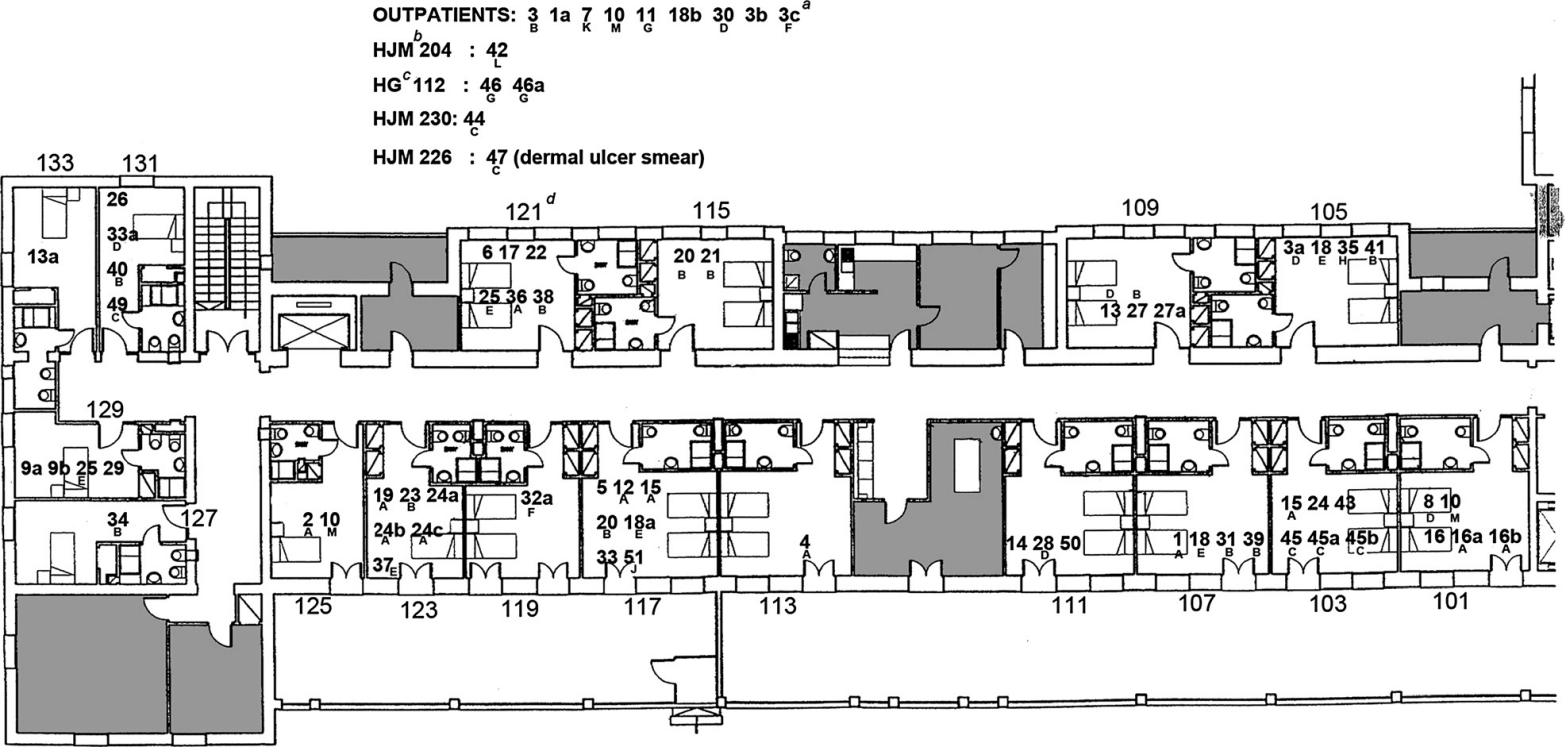
Map of Respiratory ward—Hospital Joan March. ^a^3cF: patient number 3, its fourth consecutive isolate, genotype F. ^b^HJM 204: Hospital Joan March, second floor, room 204. ^c^HG 112: Hospital General, first floor, room 112. ^d^121: room 121, first floor, respiratory ward, Hospital Joan March.

Sixty-one *C. striatum* isolates were obtained from patients hospitalized in the respiratory ward of our hospital, nine from outpatients, three from patients hospitalized in different wards of the same hospital, and two from a patient in the Hospital General de Palma, who later became an outpatient of our hospital (Fig.1).

In 41 (56.9%) specimens, *C. striatum* isolates were in pure culture, in 19 (26.4%) they were accompanied by *Pseudomonas aeruginosa*, in six (8.3%) by *Stenotrophomonas maltophilia*, and by other bacteria in six cases (8.3%). In 44 patients a single *C. striatum* isolate was obtained, in 12 patients two were obtained, in six patients three were obtained, and in two patients four isolates were obtained; there was a trend that the more repeat positive cultures, the greater the severity of bronchial obstruction.

### Antibiotic susceptibility

All isolates showed antibiotic multiresistance, defined as resistance to three or more different antibiotic families. All isolates were resistant to ciprofloxacin, and all were sensitive to vancomycin (16% sensitive to vancomycin, exclusively). The percentage of sensitivity to the remaining antibiotics tested is shown in Table S2 (see the Supporting information).

### Identification and labelling of clones

Amplification and sequencing of the ITS1 region and the *gyrA* and *rpoB* genes in 49 *C. striatum* isolates, allowed the identification of 12 different clones whose identity was simplified by attributing a capital letter to each (see Supporting information, Fig. S1).

Based on the phenotypic data, nine phenotypic variants were determined, and are labelled with a lowercase letter from ‘a’ to ‘k’. Twenty-two isolates (44.9%) corresponded to phenotype ‘a’.

The same procedure was performed for the antibiotic profile. The combination of susceptible/intermediate/resistant to the antibiotics tested (re-considering intermediate as resistant), gave 22 different combinations. A lowercase letter from ‘a’ to ‘z’ was also assigned to each combination. The most common were ‘a’, sensitive only to vancomycin, in eight (16.3%) isolates, and ‘b’, susceptible only to vancomycin and gentamicin, in six (12.2%) isolates (Table1 and see Supporting information, Table S2).

Considering the nine phenotypic variants, the 12 genotype combinations, and the 22 antibiotype combinations, 39 variants in the 49 isolates were obtained. Nevertheless, ten isolates had identical genotype, phenotype and susceptibility patterns in different time, space and patients (Table1).

### Temporal and geographic distribution of the clones

**Figure 2:**
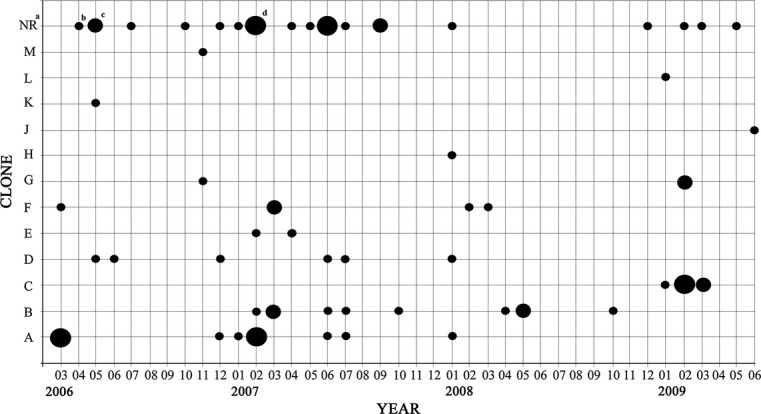
Temporal distribution of the isolates based on the genotype code. ^a^Unrecovered isolates. ^b^One isolate; ^c^Two isolates. ^d^Three isolates.

The temporal and geographic distribution of the clones was explored next (Fig.1 and 2, Table1). The isolates from different patients whose genotype suggested greater temporal and spatial relationships were the following: Clone A (patients 12 and 15, and patients 19, 24b and c) who shared rooms 117 and 123, respectively, in close time; Clone B (patients 20 and 21, and patients 31 and 39), who shared rooms 115 and 107, respectively, but not simultaneously; and Clone C, which displayed a remarkable mobility, from patient 44, to patients 45, 47 and 49, hospitalized in different rooms and wards of the hospital within 2 months.

It is worth noting that several of the isolates preserved identity, jointly in genotype, phenotype and susceptibility to antibiotics, in close time but not in spatial location, such as patients 4 and 15; 16a and b, and 36; 24b and c; 20 and 23; 28 and 31; 38 and 39; 3a and 8; and 18 and 18a.

### Respiratory colonization

In four patients (3, 18, 24 and 45) a persistent identical clone could be detected in sputum cultures separated in time. In samples corresponding to patient 3 (3 and 3c), a coincidental genotype was determined in samples obtained 23 months apart.

### Outbreaks of nosocomial infection

Some episodes of clone A direct transmission affecting three patients during March 2006 could be detected, and also of clone B in three patients between February and March 2007 (Figs.1 and 2, Table1). We also identified two small outbreaks of nosocomial infection by clone A, between December 2006 and February 2007, and by clone C between January and March 2009, corresponding to six and five isolates, respectively, each affecting four patients.

From December 2006 to July 2007, 30 strains of *C. striatum* were isolated from sputum specimens from 21 patients, but the accumulation of isolates corresponded to five different clones.

### Mortality

During our study, 25 of the 51 patients (49%) died. In 15 of the 25 deaths (60%), *C. striatum* isolates were detected without accompanying microbiota. When comparing deceased and surviving participants, mean FEV1% was 33% and 40%, mean of hospitalizations in the year before a positive culture was 2.4 and 2.1, and the average hospital stay was 80.2 days and 49.6 days, respectively (all p n.s.).

### Temperature and humidity

**Figure 3:**
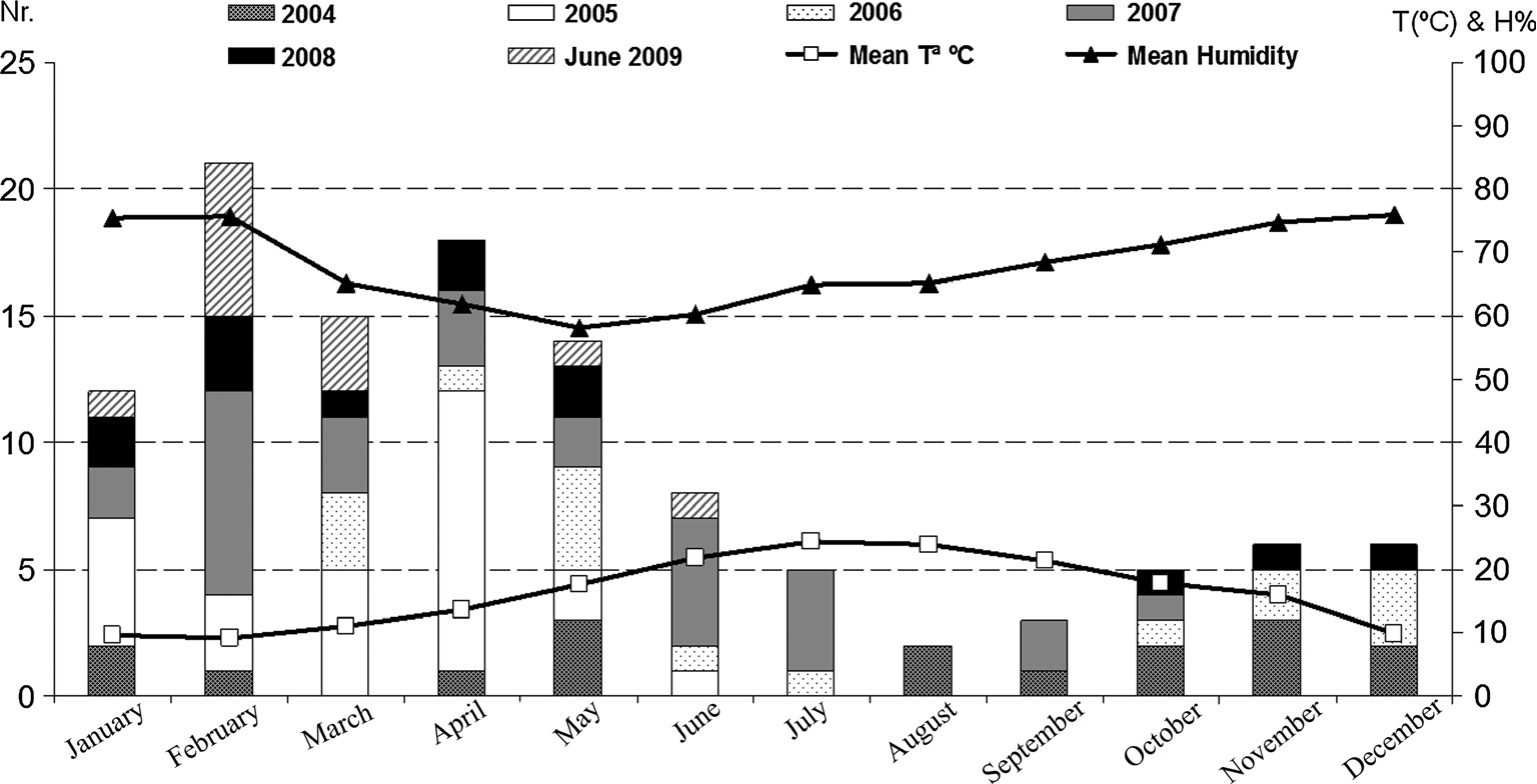
*Corynebacterium striatum* isolations versus temperature (°C) and humidity (H%); *C. striatum* isolates in Hospital Joan March, from January 2004 to June 2009.

Fig.3 represents the 115 isolates of *C. striatum* recorded in our hospital from 2004 to 2009 by month according to calendar year. The figure shows a trend to cluster cases in winter and spring, consistent with low temperatures and high humidity values.

## Discussion

In the pre-antibiotic era, Gram-positive bacteria were responsible for most severe infections; however, since the 1940s and with the introduction of penicillin, there has been an increase in Gram-negative bacteria infections until the sixth and seventh decades of the last century. Since then, a number of factors including the widespread use of broad-spectrum antibiotics for an increasing number of patients with severe chronic diseases, and the implementation of more aggressive and invasive diagnostic and therapeutic procedures have produced a resurgence of Gram-positive bacteria with multiresistance to antibiotics, generating both high morbidity and mortality. At the forefront is methicillin-resistant *Staphylococcus aureus*, but other genera have also acquired worrisome momentum, such as *Corynebacterium* spp. 5.

With *C. striatum*, a number of particularities have been repeatedly reported in a number of publications such as their high transmissibility causing nosocomial outbreaks, highlighting that most of the isolates belong to respiratory specimens, frequently associated with *P. aeruginosa* and *S. maltophilia* 7,20,21. Advanced chronic respiratory diseases, already very prevalent and expected to continue increasing in the near future, become the most susceptible conditions to infectious complications associated with this microorganism.

Outbreaks of nosocomial infection and the epidemiological determinants of *C. striatum* have been studied with the methodology available in each circumstance 15,17–22. Although pulsed-field gel electrophoresis is considered the reference standard for the identification of causative agents of nosocomial infections or outbreaks, recent publications 27,28 warn of the limitations for comparative studies of long series of isolates, the difficulty in introducing and sharing results on databases, the technical variability that hinders the reproducibility of the results, and the fact that often this method is too discriminating for a reliable determination of the phylogenetic relatedness between isolates. We chose the molecular typing method (MLSA) to identify and distinguish between clones and for comparative studies between long series of isolates. Twelve clones, nine phenotypes and 22 antibiotic susceptibility patterns were identified, establishing the following conclusions:

### Transmission

Direct transmission between patients, or through shared space, could be postulated in some cases, including the two small outbreaks of nosocomial infections caused by clone A and clone C, corresponding to six and five isolates, respectively, and each affecting four patients. However, most matches occurred in samples from patients hospitalized in rooms located at varied distances within the same ward, or even on different wards of the same hospital, suggesting that the fundamental mode of transmission is from person-to-person and through caregivers, accentuated by the wide use of inhalation devices in the treatment of respiratory diseases, as outlined in previous publications 16,18,20,21.

MLSA also allowed us to distinguish the grouping of 30 isolates of *C. striatum* in 21 patients between December 2006 and July 2007, which did not correspond to a single nosocomial outbreak but included five different clones, and to confirm that the incidence of *C. striatum* infection can be high, regardless of occasional outbreaks.

### Colonization

Clones A, B and C tended to persist for long periods, and might be isolated in the same or in different patients from 2 months up to 2 years. It is remarkable to observe jumps of several months between two identifications of the same clone, without knowing if they persisted in the habitat or in colonized patients. Thirteen patients had more than one positive culture for *C. striatum* during follow up, and the greater trend was observed with the more severe bronchial obstruction. For patient 3, three isolates were processed within 23 months. The same clone, F, and the same phenotype ‘a’, were identified in the first and third samples, varying only the antibiotype. In the intermediate sample, 3 months after the first one, we identified a different clone, named D, which was identical to the one obtained from patient 8, hospitalized at the same time in the immediately adjoining room. So, both high transmissibility and persistence could be shown in the same patient, with the additional predisposing determinants to suffering a very severe bronchial obstruction (FEV1%: 21%). Murphy *et al*. demonstrated the possibility of an identical *Haemophilus influenzae* clone persisting in the airways of the same patient even when in intermediate periods between two isolates negative sputum cultures were obtained, demonstrating that sputum cultures underestimate the frequency of *H. influenzae* colonization in the respiratory tract 29.

The relationship between accumulation of infections by an environmental pathogen such as *C. striatum* and environmental circumstances was explored with temperature and humidity values obtained at the date of each isolate, identifying a clustering during periods of low temperatures and high humidity. This observation can only have a speculative value, but we present it for comparison with future studies.

### Adaptive ability

In the 42 isolates of *C. striatum*, nine phenotypic variants were identified that, when added to the 12 molecular clones, produces 23 different combinations, showing a high adaptive variability.

### Antibiotic resistance

With regards to *C. striatum* antibiotic susceptibility, we obtained 22 different combinations, a key finding being that all isolates fulfil the criteria for multidrug-resistance. Actually, the most frequent variants were: sensitivity only to vancomycin (eight isolates, 16.3%) and sensitivity only to vancomycin and gentamicin (six isolates, 12.2%). Coincidentally with all previous publications 100% of the isolates were sensitive to vancomycin. Finally, all our isolates were resistant to ciprofloxacin.

Earlier publications 12,14,30, still described strains with broad sensitivity to antibiotics, and in fact the type strain of *C. striatum* was resistant to cefotaxime only 23. But in all later publications, nosocomial outbreaks described in intensive care areas 16,18 and other places 17,19,20,22 the multidrug resistance was a constant finding, which proves the high adaptability of *C. striatum* to antibiotic pressure. Pulmonary perfusion defects associated with bronchial obstruction in chronic respiratory illnesses can play an important role in this circumstance, making it difficult to achieve optimal therapeutic concentrations of antibiotics, which facilitates the selection of resistant strains and their persistence in the airways.

As vancomycin is the only antibiotic with sure efficacy against *C. striatum* it might pose a threat to antibiotic policies, because it competes as reserve antibiotic of choice against methicillin-resistant *Staphylococcus aureus*.

The molecular mechanisms of resistance to antibiotics of *C. striatum* are poorly understood. The plasmid pTP10, whose genetic material is capable of encoding 16 antimicrobial resistances, the acquisition of a specific transposon (Tn5432), and genes related with antibiotic resistance have already been characterized elsewhere 19,22,23,31,32. Further studies are needed to clarify the mechanisms of multidrug resistance to antibiotics in *C. striatum*.

### Morbidity and mortality

Information about *C. striatum* virulence factors is limited. In addition, their causality associated with mortality of *C. striatum* infection is highly variable and often speculative.

Regarding morbidity, there is a broad agreement on the factors predisposing to infection by *C. striatum*, as previously discussed. There is an increased frequency of repeated isolations the greater is the degree of bronchial obstruction, as measured by FEV1%.

The high mortality rates observed in our study can be partly attributed to the severity of respiratory disease, and also to the infectious exacerbation of disease by *C. striatum*. On the one hand, we noted a worse FEV1% and more admissions and hospital stays among deceased patients versus survivors. With regards to infection by *C. striatum*, the 18 deaths among the 42 patients who could complete the molecular study, were accumulated in clones A, B, D and F, and in phenotype ‘a’, but without statistical significance. However, the trend towards increased mortality in patients who suffered respiratory infections by bacterial strains with broader multidrug-resistance (four susceptible to vancomycin only, and two others to vancomycin and gentamicin) should be noted.

The main strengths of our study are the accuracy of the correct identification of the isolates involved in respiratory infections by *C. striatum*, in a homogeneous group of patients within a defined physical space, and over a long period of time to facilitate monitoring. These factors helped to follow the path of each clone and their epidemiological patterns. A major limitation is the loss of 23 unrecovered isolates obtained during this period, belonging to nine patients.

To conclude, *C. striatum* is a health concern because it is an emergent Gram-positive environmental bacterium, prevalent, highly persistent and transmissible person-to-person and through caregivers, with usual multidrug resistance, that can cause opportunistic severe infection and long-term airway colonization in patients with advanced chronic respiratory disease (older, more obstructed and poorly perfused, with frequent and prolonged hospitalizations, exposed to repeated cycles of antibiotics), and often the cause of outbreaks of respiratory nosocomial infection. Prevention should be based on guideline-based management of advanced respiratory disease and its frequent exacerbations, enforcing hygiene measures in caregivers and therapeutic devices, and treatment according to antibiogram or, should it be unavailable, with vancomycin.

## Supporting Information

**Figure S1.** Alphabetical codes for the genotype results, previously obtained (Gomila *et al*. [23]). **Table S1.** Alphabetical codes for the phenotype previously obtained (Gomila *et al*. [23]). **Table S2.** Alphabetical codes for the antibiotic susceptibility results, previously obtained (Gomila *et al*. [23]).
