# Supplementary material for: Respiratory infection by Corynebacterium striatum: epidemiological and clinical determinants
Source: New Microbes New Infect. 2014 Jun 27;2(4):106–14. doi: 10.1002/nmi2.48 (PMC4184579; doi:10.1002/nmi2.48)
Supplement: Supplementary file 1 — Figure S1. Alphabetical codes for the genotype results, previously obtained (Gomila et al. [23]). Table S1. Alphabetical codes for the phenotype previously obtained (Gomila et al. [23]). Table S2. Alphabetical codes for the antibiotic susceptibility results, previously obtained (Gomila et al. [23]). [file nmi20002-0106-sd1.docx]

Supplementary Material Figure S1. Alphabetical codes for the genotype results, previously obtained (Gomila et al. BMC Microbiology 2012)

**

**

Comprehensive information about the codes assigned from the amplification and sequencing of the ITS1 region and the *gyrA* and *rpoB* genes, can be found in Gomila et al. BMC Microbiology 2012, 12:52

Supplementary Material Table S1. Alphabetical codes for the phenotype previously obtained (Gomila et al. BMC Microbiology 2012)

| **PHENOTYPE CODE (Positive reaction = 1, Negative reaction = 2)** | | | | | | | | | |
| --- | --- | --- | --- | --- | --- | --- | --- | --- | --- |
| GLU | 1 | 1 | 1 | 1 | 2 | 1 | 1 | 1 | 1 |
| SUC | 1 | 1 | 1 | 1 | 2 | 1 | 1 | 1 | 1 |
| RIB | 2 | 2 | 2 | 1 | 2 | 2 | 2 | 2 | 2 |
| MAL | 2 | 2 | 2 | 2 | 2 | 2 | 2 | 2 | 2 |
| α-GLU | 2 | 2 | 2 | 2 | 2 | 2 | 2 | 2 | 2 |
| β-GLU | 2 | 2 | 2 | 2 | 2 | 2 | 2 | 2 | 2 |
| NAG | 2 | 2 | 2 | 2 | 2 | 2 | 2 | 2 | 2 |
| GLY1 | 2 | 2 | 2 | 2 | 2 | 2 | 2 | 2 | 2 |
| ONPG | 2 | 2 | 2 | 2 | 2 | 2 | 2 | 2 | 2 |
| PHS | 1 | 1 | 2 | 1 | 2 | 1 | 1 | 1 | 2 |
| EST | 2 | 1 | 2 | 1 | 2 | 1 | 1 | 1 | 1 |
| PRO | 1 | 1 | 1 | 1 | 1 | 1 | 1 | 2 | 1 |
| TRY | 1 | 1 | 1 | 1 | 1 | 1 | 1 | 1 | 1 |
| PYR | 2 | 2 | 2 | 2 | 2 | 2 | 2 | 2 | 2 |
| LGLY | 1 | 1 | 1 | 1 | 1 | 1 | 2 | 1 | 1 |
| LEU | 1 | 1 | 1 | 1 | 1 | 1 | 1 | 1 | 1 |
| URE | 2 | 2 | 2 | 2 | 1 | 2 | 2 | 2 | 2 |
| NIT | 1 | 1 | 1 | 1 | 1 | 2 | 1 | 1 | 1 |
| CAT | 1 | 1 | 1 | 1 | 1 | 1 | 1 | 1 | 1 |
| PIG | 2 | 2 | 2 | 2 | 2 | 2 | 2 | 2 | 2 |
| **Phenotype Code** | **a** | **b** | **c** | **d** | **e** | **f** | **g** | **h** | **k** |
| Nr. isolates | 22 | 9 | 9 | 3 | 2 | 1 | 1 | 1 | 1 |

GLU, glucose; SUC, sucrose; RIB, ribose; MAL, maltose; α-GLU, p-nitrophenyl-α-D-glucoside; β-GLU, p-nitrophenyl- β-D-glucoside; NAG, p-nitrophenyl-N-acetyl-β-D-glucoside; GLY1, p-nitrophenyl glycoside; ONPG, o-nitrophenyl-β-D-galactopyranoside; PHS, phosphatase; EST, esterase; PRO, proline-β-naphthylamide; TRY, tryptophan-β-naphthylamide; PYR, pyrrolidine-β-naphthylamide; LGLY, leucyl-glycine-β-naphthylamide; LEU, leucine β-naphthylamide; URE, urease; NIT, nitrate reductase; CAT, catalase; PIG, pigment.

Supplementary Material Table S2. Alphabetical codes for the antibiotic susceptibility results, previously obtained (Gomila et al. BMC Microbiology 2012)

| **ANTIBIOTIC SUSCEPTIBILITY CODE**  **(Susceptible = 1, Intermediate or Resistant = 3)** | | | | | | | | | | | |
| --- | --- | --- | --- | --- | --- | --- | --- | --- | --- | --- | --- |
| PEN | IMI | ERY | RIF | TET | VAN | CIP | GEN | CEF | TSX | **Code** | Nr. isolates |
| 3 | 3 | 3 | 3 | 3 | 1 | 3 | 3 | 3 | 3 | **a** | 8 |
| 3 | 3 | 3 | 3 | 3 | 1 | 3 | 1 | 3 | 3 | **b** | 6 |
| 1 | 1 | 3 | 3 | 1 | 1 | 3 | 3 | 3 | 3 | **c** | 4 |
| 3 | 3 | 3 | 3 | 1 | 1 | 3 | 3 | 3 | 3 | **d** | 3 |
| 3 | 3 | 1 | 3 | 3 | 1 | 3 | 1 | 3 | 3 | **e** | 3 |
| 3 | 1 | 3 | 1 | 1 | 1 | 3 | 1 | 3 | 3 | **f** | 3 |
| 1 | 1 | 3 | 1 | 3 | 1 | 3 | 1 | 3 | 3 | **g** | 3 |
| 3 | 3 | 3 | 3 | 1 | 1 | 3 | 1 | 3 | 3 | **h** | 2 |
| 3 | 3 | 1 | 3 | 1 | 1 | 3 | 1 | 3 | 3 | **k** | 2 |
| 3 | 1 | 3 | 3 | 1 | 1 | 3 | 1 | 3 | 3 | **l** | 2 |
| 1 | 1 | 3 | 3 | 3 | 1 | 3 | 1 | 3 | 3 | **m** | 2 |
| 3 | 3 | 3 | 1 | 1 | 1 | 3 | 1 | 3 | 3 | **n** | 1 |
| 3 | 3 | 3 | 1 | 1 | 1 | 3 | 1 | 3 | 1 | **o** | 1 |
| 3 | 3 | 1 | 3 | 3 | 1 | 3 | 3 | 3 | 3 | **p** | 1 |
| 3 | 3 | 1 | 3 | 1 | 1 | 3 | 3 | 3 | 3 | **r** | 1 |
| 3 | 1 | 3 | 3 | 3 | 1 | 3 | 1 | 3 | 3 | **s** | 1 |
| 3 | 1 | 3 | 3 | 3 | 1 | 3 | 1 | 3 | 1 | **t** | 1 |
| 3 | 1 | 3 | 1 | 3 | 1 | 3 | 3 | 3 | 3 | **v** | 1 |
| 3 | 1 | 3 | 1 | 1 | 1 | 3 | 1 | 3 | 1 | **w** | 1 |
| 1 | 3 | 3 | 3 | 3 | 1 | 3 | 3 | 3 | 3 | **x** | 1 |
| 1 | 1 | 3 | 1 | 1 | 1 | 3 | 1 | 3 | 1 | **y** | 1 |
| 1 | 1 | 1 | 3 | 1 | 1 | 3 | 1 | 3 | 3 | **z** | 1 |

PEN, penicillin; IMI, imipenem; ERY, erythromycin; RIF, rifampicin; TET, tetracycline; VAN, vancomycin; CIP, ciprofloxacin; GEN, gentamicin; CEF, cefotaxime; TSX, trimethoprim-sulfamethoxazole. Their antibiogram profiles were established by E-test assay (AB Biodisk, Solna, Sweden) on Mueller-Hinton agar plates supplemented with 5% of blood (bioMérieux, Marcy d'Etoile, France), according to CLSI recommendations.
